# How evidence-based is an 'evidence-based parenting program'? A PRISMA systematic review and meta-analysis of Triple P

**DOI:** 10.1186/1741-7015-10-130

**Published:** 2012-11-02

**Authors:** Philip Wilson, Robert Rush, Susan Hussey, Christine Puckering, Fiona Sim, Clare S Allely, Paul Doku, Alex McConnachie, Christopher Gillberg

**Affiliations:** 1 Centre for Rural Health, University of Aberdeen, Centre for Health Sciences, Old Perth Rd, Inverness IV2 3JH, Scotland; 2 Department of Health Sciences, Queen Margaret University, Queen Margaret University Drive, Musselburgh EH21 6UU, Scotland; 3 Cromarty Medical Practice, Allan Square, Cromarty, Ross-shire IV11 8YF, Scotland; 4 Institute of Health and Wellbeing, University of Glasgow, Caledonia House, Royal Hospital for Sick Children, Dalnair St, Yorkhill, Glasgow G3 8SJ, Scotland; 5 Robertson Centre for Biostatistics, University of Glasgow, Boyd Orr Building, University Avenue, Glasgow G12 8QQ, Scotland

**Keywords:** parenting, public health, child psychology, behavioral family intervention, systematic review, meta-analysis

## Abstract

**Background:**

Interventions to promote positive parenting are often reported to offer good outcomes for children but they can consume substantial resources and they require rigorous appraisal.

**Methods:**

Evaluations of the Triple P parenting program were subjected to systematic review and meta-analysis with analysis of biases. PsychInfo, Embase and Ovid Medline were used as data sources. We selected published articles reporting any child-based outcome in which any variant of Triple P was evaluated in relation to a comparison condition. Unpublished data, papers in languages other than English and some book chapters were not examined. Studies reporting Eyberg Child Behavior Inventory or Child Behavior Checklist scores as outcomes were used in the meta-analysis.

**Results:**

A total of 33 eligible studies was identified, most involving media-recruited families. Thirty-one of these 33 studies compared Triple P interventions with waiting list or no-treatment comparison groups. Most papers only reported maternal assessments of child behavior. Twenty-three papers were incorporated in the meta-analysis. No studies involved children younger than two-years old and comparisons of intervention and control groups beyond the duration of the intervention were only possible in five studies. For maternally-reported outcomes the summary effect size was 0.61 (95%CI 0.42, 0.79). Paternally-reported outcomes following Triple P intervention were smaller and did not differ significantly from the control condition (effect size 0.42 (95%CI -0.02, 0.87)). The two studies involving an active control group showed no between-group differences. There was limited evidence of publication bias, but there was substantial selective reporting bias, and preferential reporting of positive results in article abstracts. Thirty-two of the 33 eligible studies were authored by Triple-P affiliated personnel. No trials were registered and only two papers contained conflict of interest statements.

**Conclusions:**

In volunteer populations over the short term, mothers generally report that Triple P group interventions are better than no intervention, but there is concern about these results given the high risk of bias, poor reporting and potential conflicts of interest. We found no convincing evidence that Triple P interventions work across the whole population or that any benefits are long-term. Given the substantial cost implications, commissioners should apply to parenting programs the standards used in assessing pharmaceutical interventions.

See related commentary: http://www.biomedcentral.com/1741-7015/10/145

## Introduction

### Rationale

Problems in effective parenting are increasingly seen as a significant public health issue [1] and public policy has come to reflect this. The Positive Parenting Programme (Triple P) [2] is a multi-level behavioral family intervention which has been proposed [3,4] and used [5,6] in recent years on a whole-population basis as a public health intervention in addition to its use on a more targeted basis. Many administrative entities (cities or counties) throughout the world have adopted or are in the process of adopting the program on a large scale, with substantial cost implications [7]. UK National Institute for Clinical Excellence guidelines suggest Triple P is an effective educational intervention for parents of children with conduct disorder, a recommendation which carries considerable weight in policy and purchasing decisions in England [8].

The evidence base for Triple P appears to be extensive, with more than 200 publications and a large number of published randomized trials. There are four existing meta-analyses of the program [9-12], uniformly reporting positive effects on child behavior, but these reviews did not make systematic attempts to analyze risk of bias beyond the differing effect sizes attributable to different informants [9,11]. Moderators of effectiveness, such as severity of presenting problems, intensity of intervention and age/gender of the child, were assessed in three reviews [9,11,12]. There is some doubt about the effectiveness of Triple P in deprived communities [11], with lone parents [13] and among younger children, and the overall impact at population level has not been examined in detail. Much of the published work is authored by affiliates of the Triple P organization putting the independence of the evidence in a less favorable position.

We have used Preferred Reporting Items for Systematic Reviews and Meta-Analyses (PRISMA) guidelines [14] to examine reporting and other biases in a systematic way and to delineate any gaps in the evidence base supporting Triple P. We have focused on child-based outcomes in this review since the ultimate aim of parenting programs is to improve children's wellbeing.

### Objectives

We examined the published data to:

• Identify characteristics of the populations in which Triple P interventions have been subject to investigation

• Clarify which comparison conditions were used in Triple P evaluations

• Identify child-based outcome measures and which informants provided outcome data

• Examine critically the design of studies in which comparisons with alternative interventions have been reported

• Clarify any contribution of publication bias to the existing meta-analyses through examination of trial registry entries, funnel plots, and meta-regression approaches

• Clarify any contribution of outcome reporting bias and selective reporting of results in article abstracts

## Methods

### Protocol and registration

We did not register the protocol for this review.

### Eligibility criteria

Published articles in which any level of Triple P (or a precursor behavioral family intervention from the same group of authors) was used, in which any (non-Triple-P) comparison condition was employed, and in which a quantitative child-based outcome was reported, were eligible for inclusion in the systematic review. Criteria for the meta-analysis were more restrictive: eligible studies were randomized controlled trials (RCTs) reporting Child Behavior Checklist or Eyberg Child Behavior Inventory scores for intervention and comparison groups.

Journal articles published in English before September 2011 were eligible for inclusion. We also examined book chapters, whole books and electronic documents available locally and through the United Kingdom's inter-library loan system.

### Information sources

We searched databases PsycINFO 1970- August 2011, Embase 1980- August week 3 2011 and Ovid Medline 1950-August week 3 2011. We also included all journal articles, books and book chapters listed on the Parenting and Family Support Centre Triple-P database at the University of Queensland [15] (accessed 16 September 2009 and 29 August 2011) and relevant secondary references from the four available systematic reviews [9,11,12,16].

### Search

A search was carried out on 29 August 2011 using the following strategy:

Keywords = "bfi" or "Behav$ Family Intervention" or ["parenting" and "Triple"] or ["positive" and "parenting"].

### Study selection

**Figure 1:**
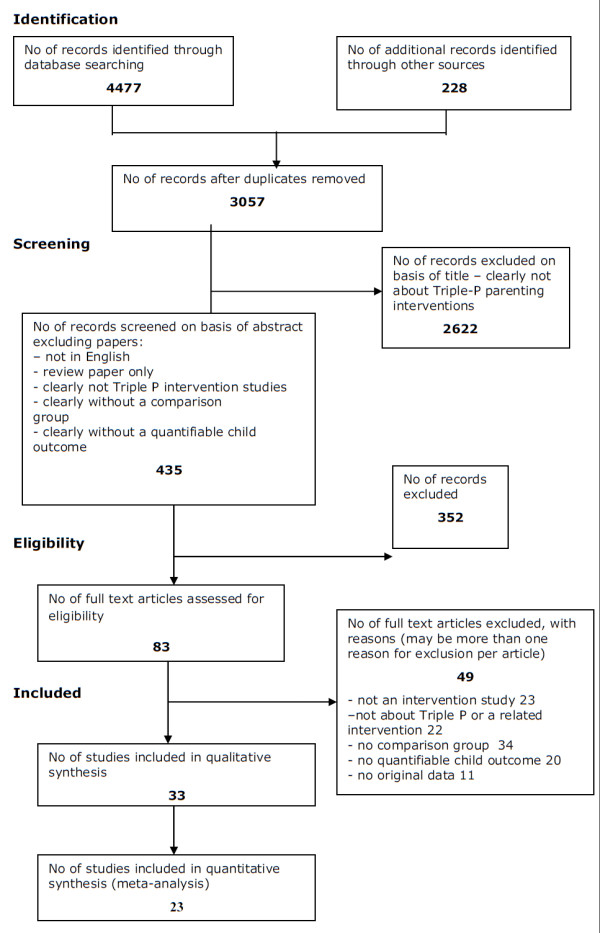
**PRISMA diagram**.

The study selection process is illustrated in Figure 1.

Screening:

In the first stage, papers which were clearly not:

• intervention studies or

• studies about the Triple-P parenting program or one of its precursors were excluded on the basis of title alone.

For the next stage papers were rejected which:

• were not published in the English language

• were not intervention studies

• were not conducted using a comparison group

• did not report a quantifiable child outcome.

In addition, review papers and book chapters which were clearly reviews were excluded.

Full documents were obtained for the remaining records.

Papers were rejected at this stage if they:

• were not intervention studies

• were not conducted using a non-Triple P comparison group

• did not report a quantifiable child outcome

• did not use Triple P or one of its precursors as an intervention

• did not report original data.

Eligible papers were tabulated and used in the qualitative synthesis.

For the meta-analysis, papers from randomized trials reporting the two most commonly used outcome measures, the Intensity scale of the Eyberg Child Behavior Inventory [17] (ECBI-I) and the Externalizing Behavior subscale of the Child Behavior Checklist (CBCL) [18] were used. These are the outcome measures reported in other meta-analyses of Triple-P child-based outcomes and are applicable to children 2- to 16-years old. Other child-based outcomes (apart from the Problem subscale of the ECBI and the Internalizing subscale of the CBCL) were reported in too few studies to allow meaningful meta-analysis. Reductions in ECBI-I and CBCL scores represent improvement. Scores on the ECBI and CBCL subscales not reported here generally mirrored those that we have reported but effect sizes were usually of lesser magnitude.

### Data collection process

Data were collected, with permission, onto a form based on that used by the Scottish Intercollegiate Guideline Network [19] (accessed 11 October 2012). For each paper, two of the authors completed the data collection form. If authors disagreed, a third author adjudicated. As our analysis concerned only published data, we did not seek to obtain further data from investigators.

### Data items

The following variables were assessed:

• Numbers of patients or families included in the study

• Main characteristics of the patient population

• Nature of the intervention being investigated

• Which outcomes were compared across groups

• Nature of the control or comparison group

• Length of follow-up

• Nature of child-based outcome measure(s) used in the study

• Which outcomes were reported in article Results and Abstract sections

• Whether a principal outcome measure was pre-specified

• Whether a power calculation was included

• Whether the assignment of subjects to treatment groups was randomized

• Whether an adequate concealment method was used (RCTs only)

• Whether reporters of the child-based outcomes were blind to treatment allocation

• Whether treatment and control groups were similar at baseline

• Dropout rates for participants recruited into each arm of the study

• Whether group differences were analyzed by intention to treat

• Whether subgroup analyses were performed

• Mean and standard deviation of post-intervention child-based outcome measures (for meta-analysis)

• Whether a statement of study funding was included

• Affiliations of authors

• Whether a conflict of interest statement was included

• Whether trials were registered with a public trials registry.

### Risk of bias in individual studies

Outcome reporting bias within eligible studies was reported qualitatively. Numerical summaries were made of the likelihood of statistically significant and non-significant results being equally reported in the Results and Abstract sections of published papers.

### Summary measures

The effect size (ES) for each study included in the meta-analysis was estimated using the standardized mean difference (SMD), with post-intervention mean and pooled standard deviation. Hedges g, under a random effects modelling approach, was used to obtain unbiased estimates of ESs. From studies with more than one treatment group, or subgroups reported separately, an averaged effect was derived based on sample size, standard deviation and mean.

### Synthesis of results

Both fixed and random effects models were generated, but the resulting models were very similar and only the random effects model is reported here. Random effects models assume that treatment effects may differ between studies, and this assumption has face validity given that treatment intensities and types of participants varied between studies.

Variation in SMDs attributable to heterogeneity was assessed with the I-squared statistic (that is, the percentage of between study heterogeneity attributable to variability in the true treatment effect, rather than sampling variation).

### Risk of bias across studies

Publication bias was assessed with funnel plots which illustrate the possibility of selective publication of small studies with positive results. Egger's regression based adjustment method was used on the data presented on funnel plots.

### Additional analyses

We planned sensitivity analyses in relation to authorship (Triple-P affiliated versus non-Triple-P affiliated). We also planned a subgroup analysis of data obtained on child behavior from sources other than the mother or principal carer (for example, fathers, teachers, independent observers).

In order to assess whether baseline symptom severity moderated treatment effects, we undertook a random effects meta-regression to investigate the association of baseline (pre-intervention) values with ES, examining only those studies which employed the most commonly used outcome measure - the ECBI Intensity scale score.

## Results

### Study selection

The selection process is illustrated in Figure 1.

### Study characteristics

The main characteristics of the studies are presented in additional file 1. Most of the studies (26/33) used a waiting list control condition where treatment was offered immediately after the post-intervention assessment. This design precluded control group follow-up beyond the end of the intervention. Comparisons of intervention and control groups beyond the duration of the intervention were only possible in five studies.

### Risk of bias within studies

Data on the risk of bias of each study are presented in Table 1.

**Table 1:** Risk of bias in individual studies.

Paper	Blinding of assessors?	Treatment and control groups similar at baseline?	Percentage drop out at post intervention measure?	Analyzed by intention to treat	Subgroup analyses reported?	Statement of study funding	Included in meta-analysis?
Bodenmann *et al. *[32]	No	Yes	Triple P 5% CCET 8% Control 23% (at long term follow up)	No	Yes	Yes. Gebert Ruef Foundation (Switzerland)	Yes
Connell *et al. *[50]	No	More females in control group	Intervention 0% Control 8%	No	Yes	No	Yes
Gallart & Matthey [26]	No	Yes (not tabulated)	Not stated (9% overall)	No	Yes	No	Yes
Hahlweg *et al. *[51]	No	Yes	Intervention mothers 14% Control mothers 3% All fathers 19% (unable to distinguish intervention & control attrition)	No	No	Yes. Deutsche Forschungsgemeinschaft	Yes
Hahlweg *et al. *[13] (data also reported in [52])	No (parents and teachers) Yes (observers)	More parents in control group were single in comparison to the intervention group: 34% and 15.6%, respectively. Baseline differences between groups for two-parent households	Intervention 0.5% Control 1%	Yes	Yes	Yes. Deutsche Forschungsgemeinschaft	Yes
Hoath & Sanders [53]	No (parents) Not known (teachers)	Control group had lower family income	Intervention 10% Control 0%	No	No	No	Yes
Joachim *et al. *[54]	No	Higher proportion of male children in control group	Intervention 15% Control 10%	Yes	Yes	No	Yes
Leung *et al. *[55]	No	Yes	Intervention 28% Control 20%	Yes - but only per protocol results tabulated	No	No	Yes
Markie-Dadds & Sanders [56]	No	Yes	Intervention 3% Control 0%	No	Yes	No	Yes
Markie-Dadds & Sanders [57]	No	Yes	Intervention 28% Control 23%	Yes - but only per protocol results tabulated	Yes	Queensland Health and the National Health and Medical Research Council	Yes
Martin & Sanders [58]	No	Treatment group had lower ECBI scores	Intervention 30% Control 50%	No	Yes	No	Yes
Matsumoto *et al. *[59]	No	Yes	Intervention 0% Control 0%	Yes (in effect)	No	No	Yes
Matsumoto *et al. *[60]	No	No. ECBI scores substantially higher in intervention group	Not stated	No	No	No	Yes
McTaggart & Sanders [21]	No	Yes	Not known	No	Yes	No	Not ECBI/CBCL
Morawska & Sanders [61]	No (parents) Yes (observers)	Yes	Intervention 12% Control 10%	Yes - but only per protocol results tabulated	Yes	No	Yes
Morawska & Sanders [62]	No	No. ECBI scores substantially higher in intervention group	Intervention 11% Control 3%	Yes - but only per protocol results tabulated	Yes	Yes. Telstra Foundation.	Yes
Morawska *et al. *[63]	No	Yes	Intervention 18% Control 18%	Yes - but only per protocol results tabulated	Yes	No	Yes
Nicholson & Sanders [28]	No (parents and step parents), Possibly (teenager's self-report)	Yes	40% therapist-delivered 45% self-delivered 5% waiting list control	No	yes	Yes. National Health and Medical Research Council	Not ECBI/CBCL
Plant & Sanders [64]	Yes (video observations) No (parent report)	Yes	Nil in all three groups	Yes (in effect)	Yes	Yes. Australian Research Council and Apex Foundation	ECBI only used as entry screener
Prinz *et al. *[5]	Not clear	Not clear (five year average data presented)	Not known	Yes (in effect)	No	Yes. US CDC	Not ECBI/CBCL
Roberts *et al. *[33]	Yes (video observations) No (parent report)	In some scales	37% intervention 35% control	No	Yes	Yes. Western Australian Health Promotion Foundation	Not ECBI/CBCL
Sanders *et al. *[65]	Yes (video observations) No (parent report)	No data presented	EBFI 23%; SBFI 17% SDBFI 18%; control 8%	No	Yes	Yes. Grants from Queensland Health and the National Health and Medical Research Council	Yes
Sanders *et al. *[66]	No	Yes	Not stated	Not clear	Yes	Partial - acknowledged source of TV programs and funding for distribution of video material	Yes
Sanders *et al. *[6]	No	No. Intervention area sample younger, poorer, less well educated and more likely to be single	Not applicable	Not applicable	Yes	Yes. Several funders	Not ECBI/CBCL
Sanders *et al. *[27]	No	No data presented except baseline measures	Intervention 23% Control 12%	Yes	No	Yes. Australia Research Council	Yes
Stallman & Ralph [25]	No (parents) Possibly (teenager's self-report)	Yes	Intervention 19% Control 11%	Yes, but only per protocol results tabulated	Yes	Yes. Australian Rotary Health Research Fund, grant	Not ECBI/CBCL
Turner *et al. *[67]	No	Yes	Intervention 23% Control 28%	No	Yes	Yes. Queensland Health and Queensland Department of Premier and Cabinet	Yes
Turner & Sanders [68]	Yes (video observations) No (parent report)	Yes	Intervention 19% Control 14%	For measures with a significant univariate condition effect at post-assessment	Yes	No	Yes
Turner *et al. *[29]	Yes (video observations) No (parent report)	Yes	Intervention 0% Control 11%	No	Yes	Yes. National Health and Medical Research Council of Australia	Not ECBI/CBCL
West *et al. *[22]	No	Yes	Intervention 21% Control 6%	Yes	Yes	Yes. Telstra Foundation	Not ECBI/CBCL
Whittingham *et al. *[24]	No	Yes	Intervention 0% Control 10%	Yes	Yes	Yes. School of Psychology University of Queensland	Yes
Wiggins *et al. *[23]	No	Yes	Intervention 10% Control 26%	Yes	Yes	No	Yes
Zubrick *et al. *[20]	No	No. Intervention area sample had younger children, less highly educated parents, more parenting problems and higher child ECBI scores. Different recruitment methods in intervention and control areas	Intervention 14% Control 4%	Not applicable	Yes	Yes. Western Australian Department of Health	No - Not randomized, and uncorrected outcome data for control group not given

CBCL, Child Behavior Checklist; CCET, Couples Coping Enhancement Training; EBCI, Eyberg Child Behavior Inventory; EBFI, Enhanced Behavioural Family Intervention (level 5); SBFI, Standard Behavioural Family Intervention (level 4); SDBFI, Self-directed Behavioural Family Intervention (level 4).

No studies were registered with national or international trials registries. All the studies apart from two [6,20] used individual or cluster [5,13,21,22] random assignment to the study group, but the mechanism of randomization was only reported in the minority of studies. No papers reported a pre-specified principal outcome measure, and no power calculations based on specific outcome measures were reported. Four papers reported a power calculation based on a general ES [23-26]. All eligible papers appeared to be co-authored by a Triple-P affiliated author, apart from one [26], although it was difficult to establish affiliation in some cases. We were, therefore, not able to conduct sensitivity analyses in relation to authorship. Conflict of interest statements were found in two papers: one [13], where 'no conflict' was reported and another [27] where royalty payments to authors were mentioned.

There is substantial risk of outcome reporting bias. Between papers, there is inconsistent reporting of subscale results within the Strengths and Difficulties Questionnaire, the ECBI, the Family Observation Schedule and the Developmental Behavior Checklist. In some papers all subscales are reported, in others selected subscales and in two, [25,28] no subscales are reported. Such selective reporting might increase the likelihood of presentation of findings supporting a favored hypothesis and the omission of less favorable analyses. Before- and after- data from the intervention group were usually presented more prominently than between-group comparisons, and this often obscured interpretation of group effects. In the 33 papers tabulated above, all except one [29] report at least one statistically significant positive child-based outcome for Triple P compared to the control condition in the Results sections, while 25/33 papers report at least one statistically non-significant result. Only 4/33 abstracts report any negative findings, whereas 32/33 report positive findings so that abstracts tend to give a more favorable picture of the effects of Triple P interventions than are supported by the more detailed findings.

### Risk of bias within studies - whole-population ('public health') interventions

Three whole-population studies met our inclusion criteria. The South Carolina study [30] was a well-designed cluster randomized trial, but the presentation did not comply with the recommended Consolidated Standards for Reporting Trials (CONSORT) format for the reporting of cluster randomized trials [31], making an accurate assessment of the implications of the paper difficult. Although it claimed to have achieved a reduction in the incidence of episodes of child maltreatment [5], it actually demonstrated an unexplained rise in reports in control areas rather than a drop in Triple P intervention sites. The description of the random allocation was poor, and the analysis was simplistic, being a two-sample t-test of county-wide measures. In particular, although some form of stratification or matching was used (it was not clear exactly how this had been done), there was no evidence that this had been accounted for in the analysis. For example, if counties were randomized within pairs, then the within-pair differences in the changes from baseline would have been of interest, but these were not reported. Therefore, although there are positive conclusions from this study, some doubt remains as to their validity.

There are two other whole-population Triple P evaluations involving a comparison group. Sanders *et al. *[6] reported a quasi-experimental study in parts of Brisbane, Sydney and Melbourne. There were substantial baseline differences between intervention and control populations. Approximately 3,000 parents were interviewed before and after the intervention, but different samples were used in each data collection and so it is not possible to characterize changes in individuals over time. Results are reported only as proportion of children with 'clinically elevated' scores rather than mean or median results. The positive child-based outcomes reported with this approach, from seven possible outcomes, were in the emotional and total problems domains of the Strengths and Difficulties Questionnaire, although neither finding would have attained conventional levels of statistical significance had allowance for multiple comparisons been made. We consider that this study offers relatively little support for any effect of triple P on children at the whole-population level.

Zubrick *et al. *[20] reported a further quasi-experimental study in two areas of Western Australia. There were again substantial differences in the characteristics of intervention and control populations. Recruitment methods differed significantly between the two areas: in the intervention area parents volunteered for active participation whereas in the control area parents volunteered to take part in a health services survey of child behavior. Analysis using hierarchical linear modelling suggests a short term improvement in ECBI externalizing behavior scores but given the potential for confounding by factors such as parental motivation it is difficult to confirm that this difference is attributable to the intervention.

### Results of individual studies

The 23 papers listed in Table 2 and associated data were used in the meta-analysis. These papers report the randomized trials in which the principal carer (usually the mother) of the index child returned ECBI or CBCL data before and after the intervention. Insufficient information is presented in most publications to allow use of intention-to-treat data, so non-imputed data for study completers only are used here.

**Table 2:** Papers included in the meta-analysis.

	**Pre-intervention**	**Post-intervention**					
			
**Author**	**Pooled baseline score**	**n1**	**mean1**	**sd1**	**n2**	**mean2**	**sd2**
		
Bodenmann *et al. *[32]^a^	118.1	50	115.4	22.6	50	104.7	23.9
Connell *et al. *[50]	157.0	12	159	10.58	12	117.33	22.77
Gallart & Matthey [26]	N/A	17	137.1	34.8	17	112	31.7
Hahlweg *et al. *[51]*	13.2	31	13	7.6	32	7.8	5.7
Hahlweg *et al. *[13]*	11.6	57	9.3	6.6	169	10.43	7.43
Hoath & Sanders [53]	162.1	11	148.36	40.29	9	125.22	35.63
Joachim *et al. *[54]	129.5	18	130.17	27.75	22	109.41	27.36
Leung *et al. *[55]	134.7	36	136.45	27.3	33	107.28	31.03
Markie-Dadds & Sanders [56]	148.6	12	146.92	15.53	28	116.3	31.53
Markie-Dadds & Sanders [57]	132.7	22	136.23	31.62	21	100.76	29.9
Martin & Sanders [58]	130.3	11	126.09	28.11	16	99.88	22.39
Matsumoto *et al. *[59]	106.3	25	105.8	25.28	25	94.12	23.79
Matsumoto *et al. *[60]	112.7	26	107.04	29.25	25	104.12	24.45
Morawska & Sanders [61]	120.7	37	123.4	27.54	75	108.59	22.96
Morawska & Sanders [62]	118.1	34	111.71	28.8	32	103.38	25.67
Morawska *et al. *[63]	146.8	27	152.26	27.14	23	124.7	20.61
Sanders *et al. *[65]	152.8	71	136.79	28.42	184	113.32	29.53
Sanders *et al. *[66]	115.9	28	108.59	33.36	28	98.74	28.04
Sanders *et al. *[27]	121.7	40	119.31	25.8	33	111.77	30.87
Turner & Sanders [68]	128.8	13	112.25	20.50	12	114.08	22.69
Turner *et al. *[67]	140.6	18	130.74	33.97	20	124.14	31.71
Whittingham *et al. *[24]	143.1	30	148.63	30.33	29	121.4	25.28
Wiggins *et al. *[23]*	65.1	22	63.4	10.4	27	57.7	9.7

Pooled mean pre-intervention score and post-intervention scores: n1, mean1 and sd1 are respectively group size, mean and standard deviation for the control groups post-intervention, and n2, mean2 and sd2 are the corresponding figures for the Triple-P intervention groups. Means and standard deviations are for ECBI-I subscale data, apart from the three papers marked with an asterisk, where the CBCL-E was reported. ^a^ECBI subscale data reported in [32] were assumed to have been transposed, and are corrected here. In this paper attrition rates at the post-treatment assessment are unknown and we assumed they remained constant. CBCL-E, Child Behavior Checklist - Externalizing Scale; ECBI-I, Eyberg Child Behavior Inventory - Intensity scale.

Only two studies [29,32] compared a Triple-P intervention with an active comparison condition - a marital distress prevention program (Couples Coping Enhancement Training, n = 50 per group) [32] and standard dietary education (group n = 12 and 9) [29]. No significant differences between the active intervention groups in terms of maternal or paternal reports of child behavior were reported in either study.

The whole population studies were excluded from the meta-analysis on the grounds of non-randomized design or the nature of the reported outcome measures.

### Synthesis of results

**Figure 2:**
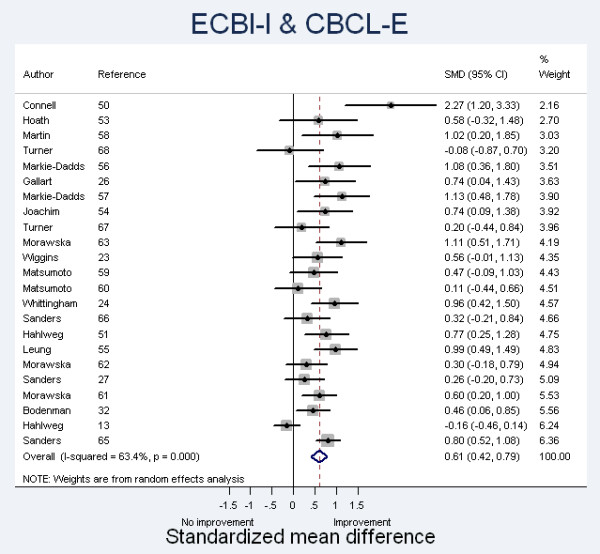
**Forest Plot (Hedges) of Standardized Mean Differences**. Studies reporting data based on ECBI or CBCL questionnaires completed by mothers are presented in increasing order of weight to final estimate, based on sample size. CBCL, Child Behavior Checklist; ECBI, Eyberg Child Behavior Inventory.

The forest plot (Hedges) depicting the included studies (maternal report ECBI-I or CBCL-E) is shown in Figure 2.

For the (generally) maternally-reported ECBI-I and CBCL-E, the summary ES was 0.61 (95%CI 0.42, 0.79) under the definition of a random-effects model with Hedges correction. Thirteen of the studies showed a significant positive effect while ten did not, with most ESs falling in the range 0.3 to 1.0.

There is evidence of heterogeneity (chi-squared = 60.16 (d.f. = 22) *P *= 0.000), with the variation in SMD attributable to heterogeneity (I-squared) = 63.4%. This level of heterogeneity indicates that there are significant differences between the studies which cannot be explained by random variation.

### Risk of bias across studies

**Figure 3:**
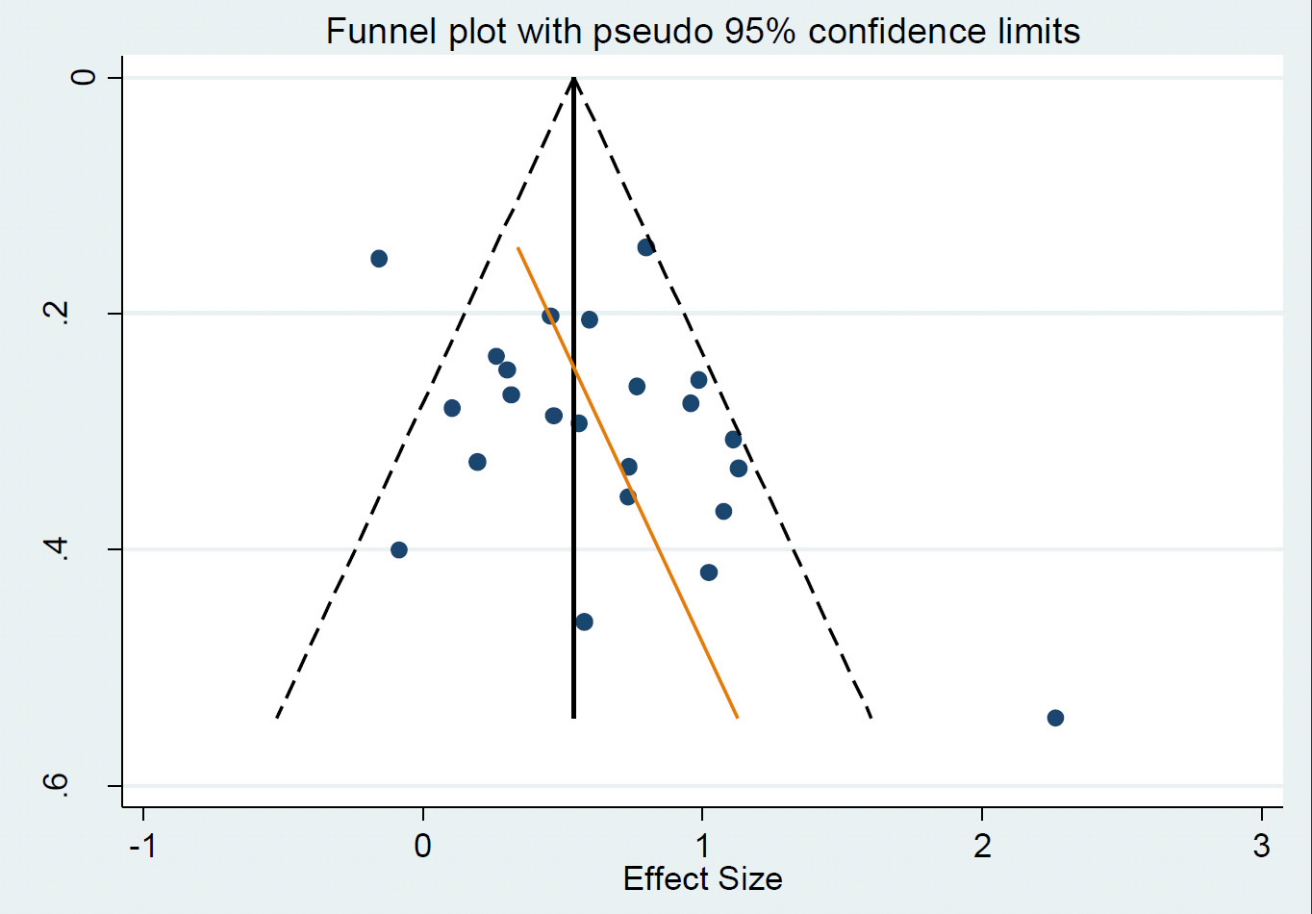
**Funnel plots for the random effects model (Hedges) based on maternally-reported ECBI-I or CBCL-E data**. CBCL, Child Behavior Checklist; ECBI-I, Eyberg Child Behavior Inventory - Intensity scale.

Publication bias was assessed by the use of a funnel plot, which illustrates the relationship between sample size and ES. Publication bias is present when there is selective reporting of small studies with positive results. Larger studies are more likely to be published successfully regardless of ES. The results are shown in Figure 3.

Egger's test (regression of the standard normal deviate of intervention effect estimate against its standard error) yielded limited evidence of small-study effects (*P *= 0.067), with an estimated bias coefficient of 1.98.

### Additional analysis

Sensitivity analysis in relation to author affiliation was not possible because of the small number of articles published without Triple-P affiliated authorship.

**Figure 4:**
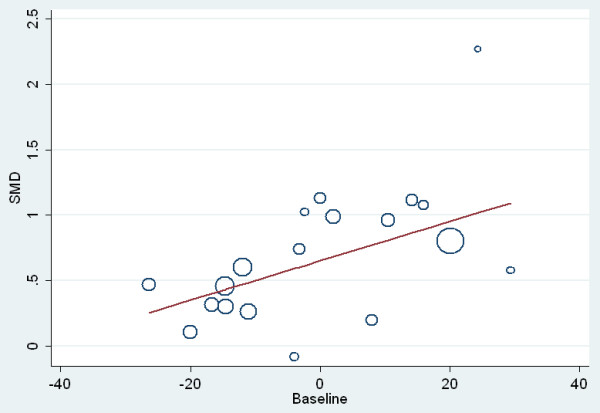
**Bubble plot of standardized between-group mean difference (SMD - equivalent to effect size) against pre-intervention (baseline) pooled ECBI-I scores**. The baseline ECBI-I scores are centered on the mean value across all included studies. The size of the circle represents the study sample size. ECBI-I, Eyberg Child Behavior Inventory - Intensity scale.

The meta-regression comparing baseline severity scores with ES for those studies which employed the ECBI-I outcome is shown in Figure 4. Ninety four percent of the between-study variance is explained by the covariate mean baseline value, and a ten-point increase in baseline ECBI-I score is associated with a 0.15 increase in ES (95% CI: 0.005, 0.025). The mean baseline ECBI-I score was 132.7, and the ES for the studies included in this meta-regression was 0.65.

Summaries of child-based outcomes reported by informants other than the principal (usually maternal) carer are reported in Table 3.

**Table 3:** Child based outcomes reported by informants other than the child's mother.

Paper	Number of children/ informants	Informant	Nature of child-based outcome measure(s)	Significance (*P *<0.05) of improvement with intervention versu control
Bodenmann *et al. *[32]	150	Father	ECBI	Not significant (Intensity and Problem subscales)
Connell *et al. *[50]	23	Father	ECBI	Significant benefit (Intensity and Problem subscales)
		Father	PDRC - Parent diary record checklist	Not significant
Hahlweg *et al. *[51]	43	Father	Child Behavior Checklist--Parent Report (CBCL 1½-5, German version)	Not significant
		Father	SDQ	Not significant
Hahlweg *et al. *[13]	198	Father	CBCL	Not significant
	273	Observers	Revised Family Observation Schedule (FOS-RIII).	Not significant
	177	Teachers	Caregiver Teacher Report Form (C-TRF 1.5 - 5)	Not significant
Hoath & Sanders [53]	21	Teachers	Sutter-Eyberg Student Behavior Inventory-Revised (SESBI-R)	Not significant
		Teachers	Child Attention Problems Rating Scale (CAP)	Not significant
Markie-Dadds & Sanders [56]		Father	ECBI	Not significant
		Father	Parent Daily Report	Not significant
Morawska & Sanders [62]	75	Teacher	Strengths and Difficulties Questionnaire	Not significant apart from hyperactivity subscale
McTaggart & Sanders [21]		Teacher	Sutter-Eyberg Student Behavior Inventory (SESBI)	Not significant (except when baseline adjustment used)
Morawska & Sanders [61]	73	Father	ECBI	Not significant
		Observers	Family observation schedule	Not significant
Nicholson & Sanders [28]	42	Self report	Child Depression Inventory	Not significant
		Self report	Child Manifest Anxiety Scale	Not significant
		Self report	Coopersmith Self-Esteem Inventory	Not significant
Plant & Sanders [64]	74	Independent observers	Revised Family Observation Schedule (FOS-RIII).	Significant benefit on negative behavior subscales (other subscales not reported)
Prinz *et al. *[5]	Approximately 170,000	Child Protective Services	Numbers of substantiated child maltreatment cases	Significant benefit^a^
		Foster Care System	Out of home placements	Significant benefit^a^
		Hospitals	Hospital visits for maltreatment	Significant benefit^a^
		Child Protective Services	Maltreatment investigation	Not significant
Roberts *et al. *[33]	23	Father	Total behavior problem subscale score of Developmental Behavior Checklist Parent Version.	Not significant
	32	Observer	FOS-IIIR noncompliance - targeted	Not significant
		Observer	FOS-IIIR noncompliance - general	Significant benefit
		Observer	FOS-IIIR Oppositional Behavior - targeted	Significant benefit
		Observer	FOS-IIIR Oppositional Behavior - general	Not significant
		Observer	FOS-IIIR Appropriate Behavior - targeted	Not significant
		Observer	FOS-IIIR Appropriate Behavior - General	Not significant
Sanders *et al. *[65]	255	Father	ECBI	Significant benefit
		Father	Parent Daily Report	Significant benefit
		Observer	Revised Family Observation Schedule (FOS-RIII). Composite score for negative child behavior	Not significant
Stallman & Ralph [25]	36	Teenagers	Conflict behavior questionnaire	Not significant
Turner & Sanders [68]	25	Independent observers	Family Observation Schedule (disruptive behaviors)	Not significant
Turner *et al. *[29]	21	Father	Child behavior checklist	Not significant
		Observer	Mealtime observation	Not significant
		Observer	Anthropometric measures	Not significant

^a^Method of analysis not clearly specified. Reported as two-sample t-tests comparing intervention and control counties, presumably of the differences between pre- and post-randomization outcome variables. However, a stratified randomization was used, so the within-pair differences in the change from baseline should be analyzed, though these are not reported. CBCL, Child Behavior Checklist; ECBI, Eyberg Child Behavior Inventory; SDQ, Strengths and Difficulties Questionnaire.

Independent observers reported benefit attributable to Triple P on at least one subscale of an observational measure in two of seven papers in which these data are reported. Teachers reported benefit in one subscale score in one of four papers with relevant data. Seven papers yielded data on paternally reported ECBI-I or CBCL-E. Summary data are reported in Table 4.

**Table 4:** Papers giving paternally-reported ECBI Intensity scores.

Author	Significantbenefit (*P *<0.05)?	n1	mean1	sd1	n2	mean2	sd2
Bodenmann *et al. *[32]^a^	No	50	109.2	18.5	50	110.1	25.2
Connell *et al. *[50]	Yes	11	154.55	17.44	12	111.0	12.41
Hahlweg *et al. *[51]*	No	16	10.7	7.0	18	7.7	5.1
Hahlweg *et al. *[13]*	No	57	9.3	7.2	141	10.2	6.9
Markie-Dadds & Sanders [56]	No	NOT REPORTED					
Morawska & Sanders [61]	No	24	111.57	20.41	49	106.07	24.37
Sanders *et al. *[65]	Yes	71	127.34	22.39	184	113.13	27.34

^a^ECBI subscale data reported in [32] were assumed to have been transposed, and are corrected here. In this paper attrition rates at the post-treatment assessment are unknown and we assumed they remained constant. n1, mean1 and sd1 are, respectively, group size, mean and standard deviation for the control groups, and n2, mean2 and sd2 are the corresponding figures for the Triple-P intervention groups. Means and standard deviations are for ECBI-I subscale data, apart from the two papers marked with an asterisk, where the CBCL-E was reported. No paternally-reported data are tabulated in Markie-Dadds and Sanders [47], but there is a statement that 'Analyses of father-reported measures of child behavior failed to produce any significant effects.' CBCL-E, Child Behavior Checklist - Externalizing scale; ECBI-I, Eyberg Child Behavior Inventory - Intensity scale.

There was strong evidence of heterogeneity (chi-squared = 29.72 (d.f. = 5) *P *< 0.001), with a variation in SMD attributable to heterogeneity (I-squared) of 83%. The summary ES for the six studies for which data were presented was 0.42 (95%CI -0.02, 0.87) under the definition of a random-effects model. The remaining study reporting non-significant results could have influenced this estimate in either direction.

## Discussion

There are a large number of published evaluations of Triple-P parenting interventions and we were able to identify 33 English-language studies which measured a child-based outcome and which compared Triple P interventions with a comparison condition. Most of the studies involved families who responded to media advertisements. These families clearly have children whose parents are finding difficulties with their behavior but may well not be typical of such families in the population. They are more likely to be motivated and literate and are sufficiently confident to present for treatment as volunteers. These characteristics would be likely to lead to high levels of compliance with treatment and better than average treatment response. Only five studies [5,6,21,33,34] did not rely upon self-referral by potential participants. All the studies involved only children over two years old. There are many forms of the Triple-P program [2], and new versions emerge regularly, but for simplicity we have not distinguished between the levels of intervention. Nevertheless, most of the studies reported on the effectiveness of small group-based Triple-P interventions, usually level 4 or 5, and synthesis of results from other Triple P levels would be limited by low numbers.

Most of the studies were relatively small and the great majority used a waiting-list control design in which the participants on the waiting list were offered active treatment immediately after the post-intervention data collection. It is, therefore, not possible to draw conclusions about the longer term effectiveness of Triple P relative to a comparison condition. Before- and after- data from the intervention group were usually presented more prominently (and frequently) than between-group comparisons, and this method of reporting often obscured interpretation of group effects and tended to increase the impression of a positive effect from the intervention. Only two trials used an active comparison group, and neither of these showed any advantage for Triple P in terms of child-based outcomes. Trials with waiting list controls or usual care provide intervention estimates which reflect the combined specific and non-specific effects that will accrue in practice, and are more likely to show between-group differences than trials with active controls [35].

A range of child-based outcome measures was used but the most commonly reported was the ECBI, completed by parents. In the majority of cases (31/33) the main informant was the mother, and these data were synthesized in our main meta-analysis. Despite some differences in methodological approach, the ES obtained in our meta-analysis of maternally-reported ECBI scores (0.61) is impressive and is broadly in line with that reported by other authors [9,11,12].

There is some evidence of publication bias from our analyses of the published work and there is additional evidence that results of a number of evaluations of Triple-P have not been published [30,36]. Further evaluation of publication bias is not possible given the uniform lack of Triple-P trial registration. Thus, despite the apparent consistency of more recently published work, there is still the possibility that these studies represent a particularly favorable picture of Triple-P. The International Committee of Medical Journal Editors (ICMJE) agreed in 2005 that only registered trials would be considered for publication. Allowing for publication time lags, about one third of the studies in our meta-analysis pre-dated the guidance and two thirds could potentially have benefitted from adopting these recommendations.

There are considerable limitations in relying on maternal report data alone: 17 papers gave outcomes reported by informants other than mothers. Five of these studies showed a relative benefit for Triple P on one or more outcome measures compared with no treatment. Meta-analytic synthesis of paternally-reported data identified a pooled ES of 0.42 but there was significant heterogeneity and the overall effect size was not significantly greater than zero. Multiple outcomes were used in five of the six papers reporting significantly positive results: primary outcomes were not pre-specified (in common with all the reported trials), and corrections were not made for multiple comparisons. Paternal reports are often difficult to assess because of missing data which may not be missing at random. The incorporation of independent direct observations of parent and child behavior into trial design provides important confirmatory information [37,38], and seven of the Triple P trials (see Table 4) included data from independent observers. Two of these seven studies reported benefit in one or more observational subscale.

It is possible that the discrepancy between maternal and paternal (or independent) reports of child behavior may be accounted for by the fact that maternal mental state improved significantly with most Triple-P interventions [10] and this may have led to a more positive maternal evaluation of the child's behavior, reflecting more optimistic states of mind. Fathers are less likely to attend sessions and independent observers are unconnected with the intervention. Related attribution effects have been reported in relation to Triple-P [39]. It is also possible that mothers are more accurate than fathers in reporting their children's behavior difficulties. One paper [13] reported a planned subgroup analysis for lone parent families - and reported no benefit from the triple P intervention.

All of the papers considered here, with one exception [26] were authored or influenced by Triple-P affiliated personnel. This is commonly observed in the early stages of development of non-pharmaceutical interventions but readers should interpret findings accordingly, particularly when authors may gain financially from the intervention under study. Although authors of Triple P interventions receive royalty payments from sales of training and materials [27], only one of the articles we obtained declared any conflict of interest. Conflict of interest may be of particular importance in interpreting studies (such as many of those reported here) in which subgroup analyses are reported [40]. Outcome reporting bias [41] may also be an important consideration in this respect, with possible significance for the interpretation of meta-analyses [42].

Claims that whole-population parenting programs have significant impact on public health are particularly important, because these may have led to substantial commitment of public funds. We were unable to find any convincing evidence of benefit from the Triple P program in the three whole-population studies eligible for inclusion in the present review.

### Summary of evidence

Although a standard meta-analysis confirmed previous findings that mothers report improved child behavior after Triple-P interventions in comparison to a waiting list control condition, fathers and independent observers generally do not report improvements that are significantly different from those attributable to the control condition (Table 4). There is an absence of evidence of sustained benefit from Triple P interventions compared to control conditions, and no evidence that Triple P is superior to any other active intervention.

### Limitations

Given the highly specific nature of the literature search, and the multiple sources of data, we believe that we have retrieved almost all of the relevant literature. We did not synthesize papers in languages other than English. We were not able to retrieve some book chapters with titles indicating possible eligible studies, but we did not find any new data reported in the many chapters we were able to retrieve. We did not obtain potentially relevant data from studies which were conducted but not subsequently published [30,36].

## Conclusions

The studies to date give proof-of-concept that group-based Triple-P may be effective in the short term according to maternal report of child behavior but, given the high risk of bias (or unknown risk of bias when reporting is poor), they do not support the view that Triple P provides other benefits to children.

The lack of convincing evidence of benefit from whole-population interventions is in line with previous work in which no significant improvement in child-based outcomes resulted from a public health parenting program [43] and with a more recent large-scale independent evaluation of Triple P in Zurich which demonstrated no impact on child behavior [44]. Along with findings from a previous systematic review [12], the results of our meta-regression support the view that some benefit might be achieved if interventions were focussed on the families of children with more severe problems. A recent Cochrane review of parent training interventions for children with established conduct problems and those at high risk of conduct disorder [45] provides robust evidence of the effectiveness of such targeted programs. It is, therefore, likely that an effective case-finding approach combined with offers of interventions to families with identified problems may be more effective than the 'public-health' approach.

Only one of the studies [26] included in our review had no apparent conflict of interest. We are aware of two further independent evaluations of Triple P which were ineligible for inclusion in our review - one published in German [46] and one recent large scale trial [44]. Both produced negative results. These findings mirror the frequently observed failure of independent replication of positive results from a range of developer-led studies. Theoretical models developed by Eisner [47], describing the mechanisms by which conflict of interest can lead to research bias, may help to explain this phenomenon.

Given the substantial cost implications, health care providers and policymakers would be well advised to apply the same standard of evidence when purchasing behavioral interventions as they do to the purchase of pharmaceutical agents or medical devices. Compulsory clinical trial registration and full and open declaration of conflicts of interest would address many of the deficits noted in our review. Pending the implementation of such mechanisms, unproven interventions should only be carried out in the context of a robust independent evaluation.

Developers and evaluators of psychological interventions should be encouraged to adhere to the guidelines dealing with good publication practice for communicating company sponsored medical research (GPP2 [48]). Journal editors and reviewers should be encouraged to adhere to CONSORT guidelines for both text and abstracts [49]: we believe that authors who choose journals that do not adhere to these guidelines, and editors who choose not to adopt them, do the field, as well as their own work, a disservice. There should be a clear expectation that instrument subscales should be reported in full and covered even-handedly in article abstracts. Care providers and policy makers should assess the generalizability of findings from socially advantaged and volunteer samples to their own situation. There is a need for registered, large, multicenter trials, with prospectively defined, long-term outcomes and active comparison groups rather than further evaluations using waiting list control groups. Rigorous systematic reviews of parenting interventions (for example [45]) attest to the importance of including data from independent observers in trials and in reviews, both in order to reduce risk of bias and to provide more convincing data on the effects of parenting interventions. Whole-population data on child behavior, reported by multiple informants, and linked to provision of parenting interventions, may be an alternative approach to the evaluation of public health parenting programs.

## Funding

The study was unfunded, apart from a contribution made by the Gillberg Neuropsychiatry Centre to pay for statistical analysis. The funder played no part in the study design, analysis or interpretation of the data except insofar as one of the authors (CG) is employed within the Gillberg Neuropsychiatry Centre at the Sahlgrenska Academy, University of Gothenburg.

## Abbreviations

CBCL: Child Behavior Checklist; CONSORT: Consolidated Standards of Reporting Trials; ECBI-I: Eyberg Child Behavior Inventory - Intensity scale; ES: effect size; PRISMA: Preferred Reporting Items for Systematic Reviews and Meta-Analysis; RCT: randomized controlled trial; SMD: standardized mean difference; Triple P: Positive Parenting Program.

## Competing interests

PW is academic advisor to the evaluation of the Glasgow Parenting Support Framework, which has Triple P as one of its core components. AMcC provides statistical advice to this evaluation. Along with AMcC and CP, PW is a co-investigator in a NIHR funded trial comparing an antenatal Triple P intervention with another parenting program, Mellow Parenting. CP is an author of the Mellow Parenting program, which is owned by a charity, and she is employed as a trainer for this program. Other authors report no conflicts of interest.

## Authors' contributions

PW, CP, PD, FS, SH and CG reviewed the published papers, RR performed the statistical analyses and AMcC provided statistical support to the authors. PW is guarantor of this paper. All authors contributed to the drafting of the article and approved the final submission.

## Pre-publication history

The pre-publication history for this paper can be accessed here:

http://www.biomedcentral.com/1741-7015/10/130/prepub

## Supplementary Material

Additional file 1 **Main characteristics of the included studies**. Tabulation, for all eligible studies [5,6,13,20-29,32,33,50,51,53-69], of numbers and characteristics of patients or families included, whether problems were likely to be in the clinical range, inter-group comparisons made, nature of the control group, length of follow-up and nature of informants. Click here for file
