# Supplementary material for: How evidence-based is an 'evidence-based parenting program'? A PRISMA systematic review and meta-analysis of Triple P
Source: BMC Med. 2012 Nov 2;10:130. doi: 10.1186/1741-7015-10-130 (PMC3532197; doi:10.1186/1741-7015-10-130)
Supplement: Additional file 1 — Main characteristics of the included studies. Tabulation, for all eligible studies [5,6,13,20-29,32,33,50,51,53-69], of numbers and characteristics of patients or families included, whether problems were likely to be in the clinical range, inter-group comparisons made, nature of the control group, length of follow-up and nature of informants. [file 1741-7015-10-130-S1.DOC]

**Additional file 1. Main characteristics of the included studies.** S-R=self-report, P=parent, M=mother, F=father, S-P=step-parent. CCET= Couples Coping Enhancement Training, ECBI=Eyberg Child Behavior Inventory, SDQ=Strengths and Difficulties Questionnaire, FOS=Family Observation Schedule, CBCL=Child Behavior Checklist. Behaviour problems were categorised as likely to be in the clinical range if mean pre-intervention values were above published clinical cut-off scores for the relevant outcome measure.

| **Paper** | **Numbers of patients or families included in this study** | **Characteristics of patient population** | **Behaviour problems likely to be in clinical range?** | **Nature of the intervention** | **Inter-group comparisons made** | **Nature of control group** | **Length of follow-up** | **Informant** | **Nature of child-based outcome measure(s)** |
| --- | --- | --- | --- | --- | --- | --- | --- | --- | --- |
| Bodenmann et al [32] | 150 couples | Swiss couples with children aged 2-12 recruited by advertisement | No | Triple P group level 4 | Triple P Vs control  Triple P Vs CCET  CCET Vs control | 1. Marital intervention (CCET)  2. No treatment control | 1 year | Mothers  Fathers | ECBI |
| Connell et al [50] | 24 families | Rural SE Queensland children aged 2-6 within clinical range of ECBI. Volunteers recruited via media campaign. | Yes | Self-directed behavioural family intervention | Intervention Vs control | Waiting list control | 10 weeks  6 months (intervention only) | Mothers  Fathers | ECBI  PDRC - Parent diary record checklist |
| Gallart & Matthey [26] | 54 parents | Parents of children aged 2-8 years. Volunteers recruited via media campaign. | Yes | Level 4 group Triple P  Level 4 group Triple P minus telephone calls | Level 4 group Triple P Vs Level 4 group Triple P minus telephone calls Vs Control | Waiting list control | 8 weeks  6 months (intervention only) | Parents (94% mothers) | ECBI |
| Hahlweg et al [51] | 69 mothers  43 fathers  in 69 families | Volunteer parents of 3-6 year olds at 4 German school sites. Families above average income. | No | Self-directed Triple P with therapist assistance | Triple P4 + telephone support Vs control | Waiting list control | 10 weeks  6 months (intervention only) | Mothers  Fathers | CBCL 1½-5, German version  SDQ |
| Hahlweg et al [13] | 280 families | German, child age average 4.5 years. Range 3-6 years. Preschool. Volunteer parents who were paid for completion of assessments. Parents 22-47years, 78% married. 51% of mothers and 62% of fathers had High school degree. Higher participation in more affluent areas | No | Level 4 group Triple P | Group Triple P Vs control | No treatment control | 2 years | Parents (singly or jointly)  Researcher (cognitive assessment)  Blind raters (FOS)  Teacher (C-TRF) | CBCL  Kaufman Assessment Battery for  Children  FOS  Caregiver Teacher Report Form (C-TRF 1.5 - 5) |
| Hoath & Sanders[53] | 21 families | Families with children aged 5-9 with diagnosed ADHD in Queensland and NSW. Recruited via media and professional referral | Yes | Enhanced group Triple P | Enhanced group Triple P Vs control | Waiting list control | 12 weeks  25 weeks (intervention & controls who completed intervention after waiting time) | Parents (ECBI, CAP)  Teachers (SESBI-R, CAP) | ECBI  Child Attention Problems Rating Scale (CAP)  Sutter-Eyberg Student Behavior Inventory-Revised (SESBI-R) |
| Joachim et al [54] | 46 families | Brisbane families with children aged 2-6, recruited by mass media or posters.  Mainly female white Australian, 60% university graduates | Yes | Topic-specific Triple P. Brief parent discussion group, two hour group, book to read and tip sheet. | Intervention Vs control | Waiting list control | 4 weeks  6 months (intervention only) | Parents (96% mothers) | ECBI  Shopping Observation Checklist (list of difficult behaviours recorded by informant) |
| Leung et al [55] | 91 | Parents in Hong Kong with children aged 3-7 who had concerns about child behaviour or had been referred because of this | Yes | Group Triple P | Group Triple P Vs control | Waiting list control | 10 weeks | Parents (96% mothers) | ECBI  SDQ  Parent Daily Report |
| Markie-Dadds & Sanders[56] | 41 families | Families with a child aged 2-6 years in southern country areas of Western Australia. Recruited via a media campaign and posters. ECBI in elevated range for eligibility | Yes | Self-directed Triple P  Enhanced self-directed Triple P | SD-TP Vs ESD-TP Vs control | Waiting list control | 12 weeks  6 months (intervention only) | Mothers  Fathers | ECBI  Parent Daily Report |
| Markie-Dadds & Sanders [57] | 63 families | Children aged 2-5 years. Recruited via a media campaign and posters. ECBI in elevated range for eligibility | Yes | Self-directed Triple P | SD-TP Vs control | Waiting list control | 15 weeks  6 months (intervention) | Mothers | ECBI  Parent Daily Report |
| Martin & Sanders[58] | 45 | Volunteer university-employed parents with children aged 2-9 years with SDQ in behaviour problem range | Yes | Work place Triple P | Work place Triple P Vs  control | Waiting list control | 8 weeks  4 months (intervention only) | Parent (no further detail) | ECBI |
| Matsumoto et al [59] | 50 families | Japanese families living in Queensland with children aged 2-10. Recruited through the Japanese Society and/or the Japanese Supplementary School. Most parents married and highly educated | No | Group level 4 Triple P | Triple P Vs control | Waiting list control | 10 weeks  23 weeks (intervention) | Parent (no further detail) | ECBI |
| Matsumoto et al [60] | 54 families | Families living in Tokyo. Recruitment method unclear. Children aged 2–10 years). All parents married, mainly college or university educated. None of the mothers in paid employment, all fathers in full-time employment. | No | Group level 4 Triple P | Triple P Vs control | Waiting list control | Not clear | Not clear | ECBI  SDQ |
| McTaggart & Sanders[21] | 25 schools  1389 pupils | First year pupils at Brisbane primary schools | No | Information campaign and level 4 group Triple P if requested | Triple P Vs control | Waiting list control | 12 weeks  6 months (intervention) | Teacher | Sutter-Eyberg Student  Behavior Inventory (SESBI) |
| Morawska & Sanders [61] | 126 families | Volunteer parents of 18-36 month olds who answered yes to concern about behaviour query. Recruited through media campaign and nurseries | No | Toddler Triple P +/- telephone support | (Toddler) Triple P  Vs  Triple P+ telephone support  Vs  control | Waiting list control | 10 weeks  6 months (intervention only) | Mother (ECBI)  Father (ECBI)  Observers (FOS) | ECBI  FOS (blinded observers) |
| Morawska & Sanders [62] | 75 families | Families with children aged 3-10 with behaviour problems and assessed as “gifted” or accelerated progression in school. Most parents married and university educated. | No | “Gifted and Talented Group Triple P”, based on group level 4 | Gifted and Talented Group Triple P Vs control | Waiting list control | 8 weeks  6 months (intervention only) | Parents (ECBI & SDQ)  Teacher (SDQ) | ECBI (parents)  SDQ (parents and teachers) |
| Morawska et al [63] | 67 families | Brisbane families with children aged 2-5 years recruited by advertisement. 66/67 participants were mothers. Mainly married, 50% university graduates | Yes | Brief intervention – 2 hour discussion group and 2 follow up telephone contacts | Brief intervention Vs control | Waiting list control | 2 weeks  6 months (intervention only) | Parents | ECBI  Parenting Relationship  Questionnaire |
| Nicholson & Sanders[28] | 60 | Australian stepfamilies with children 7-12 years, score >40 on CBCL, disturbed behaviour >6 months. Recruited by advertisement or therapy referral | Yes | 8 Behavioural Family Intervention modules (self-directed or therapist-delivered) | Therapist-delivered Vs self-directed intervention (no significant differences noted)  Both interventions combined Vs control | Waiting list control | 10-16 weeks | Parent & Step-Parent (CBC, PDR)  Self-Report (CDI, CMAS, CSEI) | CBCL  Parent Daily Report  Child Depression Inventory  Child Manifest Anxiety Scale  Coopersmith Self-Esteem Inventory |
| Plant & Sanders [64] | 74 | Australian families. Children under 6 receiving LD services. ECBI –I > 130 or ECBI-P > 14 | Yes | SSTP – enhanced (16 session) or standard (10 session) | SSTP-E Vs. WL  SSTP-S Vs. WL  SSTP-E Vs SSTP-S | Waiting list control | 10 weeks (S),  16 weeks (E & control).  1 year FU for intervention groups only | Independent observers (FOS)  Parent (not stated which) completed DBC and CPC | 30 minute parent-child interaction videos coded with FOS-RIII for negative child behaviour  Developmental Behavior Checklist (parent complete)  Caregiving Problem Checklist (2 scales) |
| Prinz et al [5] | ~170,000 | Whole population of 18 counties in South Carolina under 8 years – rural or semi-urban | No | Levels 1 to 5 – whole system approach | Intervention Vs control areas | Not specified – non-intervention counties | 2 years | Various | Numbers of substantiated child maltreatment cases  Out of home placements  Hospital visits for maltreatment  Maltreatment investigation  Other child-based outcomes described elsewhere[30] not reported |
| Roberts et al [33] | 47 families, 51children | Children aged 2-7 years recruited through Western Australian Disability Services Commission with intellectual or adaptive functioning >2SD below age norms. Most had mild developmental delays. | Yes | Stepping Stones Triple P | Intervention Vs control | Waiting list control | 16 weeks  6 months (intervention only) | Mothers (TBPS)  Fathers (TBPS)  Observer (FOS) | FOS-IIIR subscales in both targeted and generalization settings:   - Noncompliance - Oppositional Behavior - Appropriate Behavior   The total behavior problem subscale score (TBPS) of the Developmental Behavior Checklist Parent Version. (Einfeld & Tonge, 1992). Not clear if the remaining five subscales were assessed. |
| Sanders et al [65]  (a subgroup analysis was reported elsewhere ([69]) | 305 families | Families with a 3-year-old child.  Participants recruited via community outreach campaign in three low-income areas of Brisbane. Eligibility criteria included high ECBI scores and family adversity. | Yes | EBFI Enhanced Behavioural Family Intervention (level 5);  SBFI Standard Behavioural Family Intervention (level 4);  SDBFI Self-directed Behavioural Family Intervention (level 4) | EBFI Vs SBFI Vs SDBFI Vs control | Waiting list control | 15 weeks  1 year (intervention only) | Mothers (ECBI & PDR)  Fathers (ECBI & PDR)  Observer (FOS) | Revised FOS (FOS-RIII). Composite score for negative child behaviour (percentage of intervals during which the child displayed negative behaviours)  ECBI  Parent Daily Report (PDR) |
| Sanders et al [66] | 56 | Families with children aged 2-8 years. Volunteers recruited via media campaign. | No | Level 1 Triple P | Intervention Vs control | Waiting list control | 6 weeks  6 months (intervention only) | Mothers | ECBI |
| Sanders et al [6] | 2999 interviews pre-intervention  3000 interviews post-intervention | Families of children aged 4-7 years in 10 areas of Brisbane (intervention) and 10 areas of Sydney and Melbourne (control) | No | Whole system Triple P – all five levels | Intervention Vs control | Quasi-experimental design with no intervention in control areas | 2 years | Caregiver -usually mother | SDQ  2-item global measure of child functioning |
| Sanders et al [27] | 121 | Parents recruited through workplaces in Brisbane. 72% mothers, 74% married, 62% university graduates | No | Workplace Triple P (based on enhanced level 5) | Intervention Vs control | Waiting list control | 8 weeks  12 months (intervention only) | Participating parent – usually mother | SDQ  ECBI |
| Stallman & Ralph [25] | 51 families | Queensland families with child aged 11-14, with reported or elicited concern about behaviour. Volunteers recruited via media campaign. | No | Teen Triple P +/- telephone assistance to parents | Teen Triple P + telephone  Vs  Teen Triple P  Vs  Waiting list control | Waiting list control | 10 weeks  23 weeks (intervention) | Parent (SDQ)  Teenagers (CBQ) | SDQ  Conflict behaviour questionnaire |
| Turner et al [67] | 51 | Indigenous families with children aged 1-13 presenting to 4 S-E Queensland Community Health centres requesting information or advice about child behaviour problems or developmental issues. | Yes | “Culturally tailored” level 4 group Triple P | Intervention Vs control | Waiting list control | 8 weeks  6 months (intervention only) | Caregiver – 67% mothers | ECBI  SDQ (extended) |
| Turner & Sanders [68] | 30 | Brisbane families with children aged 2-6 presenting to three community child health clinics requesting advice about child behaviour problems or developmental issues | No | Primary Care Triple P | Intervention Vs control | Waiting list control | 4-8 weeks  6 months (intervention) | Primary caregiver - 94% mothers  Independent observers (FOS) | Parent Daily Report  ECBI  Home and Community Problem Checklist  FOS |
| Turner et al [29] | 21 | Families with children 1-5 years referred to paediatric or behavioural services | Yes | Behavioural parent training | Intervention Vs control | Standard dietary education | 3-4 months | Mother (CBCL)  Father (CBCL)  Independent observers (mealtime observation) | CBCL  Mealtime observation |
| West et al [22] | 101 families | Brisbane families with children between 4 and 11 years who described their child’s body size as overweight | Not known | Group Lifestyle Triple P, a modification of Level 4 Group Triple P | Intervention Vs control | Waiting list control | 12 weeks  6 months (intervention only) | Parent (LBC)  Researcher (BMI) | Lifestyle Behaviour Checklist  Body Mass Index z-score |
| Whittingham et al [24] | 59 families | Carers of child aged 2-9 with autistic spectrum disorder diagnosed by a paediatrician. Recruited via media and organizations in Brisbane. | Yes | Stepping stones Triple P | Stepping stones treatment  Vs  control | Waiting list control | 9 weeks  6 months (Intervention only) | Parent (not specified) | ECBI |
| Wiggins et al [23] | 60 families | Parents (>90% mothers) of a child aged 4-10 years in Brisbane reporting parent-child relationship problems. Parents (most married and relatively affluent) self referred following a media campaign. | Yes | Pathways Triple P (9 weekly sessions) | Pathways Triple P Vs control | Waiting list control | 9 weeks  3 months (Intervention only) | Parent | CBCL  SDQ  Parenting Relationship Questionnaire |
| Zubrick et al [20] | 1610 | Two metropolitan areas (1 intervention, 1 control) of Western Australia. Volunteer recruitment through day centres, nurseries, public information campaign. | No | Group level 4 Triple P | Intervention Vs control | Care as usual | 2 years | One parent – usually mother | ECBI |
